# A Long-Term Survivor with Tetralogy of Fallot Treated Only with the Classical Blalock-Taussig Shunt

**DOI:** 10.1155/2018/5262745

**Published:** 2018-08-19

**Authors:** Yu Yamada, Tomoko Ishizu, Hidekazu Tsuneoka, Yutaka Eki, Hitoshi Horigome

**Affiliations:** ^1^Department of Cardiology, Faculty of Medicine, University of Tsukuba, Ibaraki, Japan; ^2^Department of Cardiology, Hitachi General Hospital, Ibaraki, Japan; ^3^Department of Pediatrics, University of Tsukuba, Ibaraki, Japan

## Abstract

The prognosis of tetralogy of Fallot (TOF) treated only with Blalock-Taussig shunt (BTS) operation is unclear. A woman with TOF underwent classic BTS operation at 10 years of age. Despite no medication, she delivered two children and worked without apparent heart failure. At 72 years of age, she complained of dyspnea on exertion and leg edema. The cardiac angiogram revealed a well-patent BTS and severely stenotic right ventricular outflow tract. Right heart catheterization showed adequately maintained pulmonary blood flow with slight pulmonary arterial hypertension. Her unexpected yet favorable outcome reaffirms the importance of structural and functional self-adaptation even with cyanosis. If she had undergone a valve-sparing corrective surgery in adolescence, much better quality of life and outcome could have been expected.

## 1. Introduction

Tetralogy of Fallot (TOF) is the most common cause of cyanotic congenital heart disease, and Blalock-Taussig shunt (BTS) operation is considered the first-step management for maintaining pulmonary blood flow in TOF patients. Complete transventricular repair of TOF is the standard surgical treatment that should be performed in infancy or early childhood [1]. Long-term survival has improved because it is associated with lower operative mortality [2, 3]. However, the prognosis of patients with TOF treated by only palliative operation remains unclear. We describe a patient with TOF treated by classic BTS operation who lived a productive life without obvious symptoms in the long term.

## 2. Case Presentation

A woman with TOF underwent left classic BTS operation by the late cardiac surgeon Professor Shigeru Sakakibara at the Tokyo Women's Medical College, Tokyo, Japan, in the 1950s when she was 10 years old. She stopped visiting the hospital a few years postoperatively because she did not have difficulty performing daily activities. Subsequently, she got married at 25 years of age and gave birth to two children at 29 years of age without special gynecological care at a local hospital. She kept working on a production line while raising her children without experiencing symptoms of heart failure.

**Figure 1:**
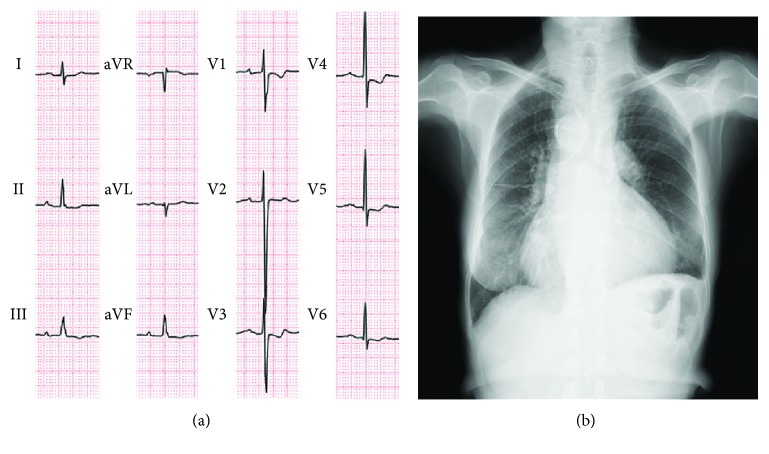
Electrocardiography and the chest X-ray. (a) Electrocardiogram shows normal sinus rhythm at 75 beats/min with a prolonged PR interval; negative T in leads II, III, and aVF; and ST depression in V1 and V4–6. (b) Chest radiograph reveals a cardiothoracic ratio of 66%, right-sided aortic arch, and dilated pulmonary arteries.

**Figure 2:**
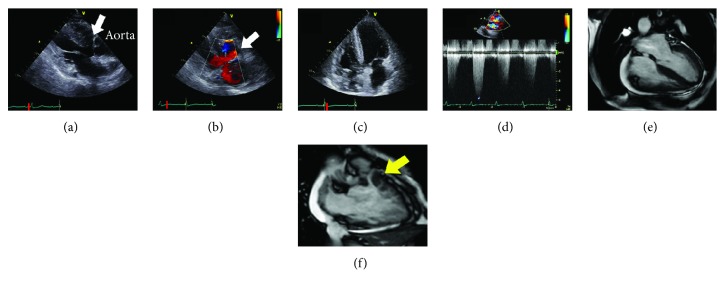
Transthoracic echocardiogram and cardiac magnetic resonance imaging (CMRI). (a) Parasternal long-axis view shows the ventricular septal defect (VSD) (white arrow) and overriding of the aorta. (b) Color Doppler image in the long-axis view shows two shunts through the VSD (white arrow). (c) Apical four-chamber view shows the hypertrophic right ventricle. (d) There is an RV outflow obstruction with a peak velocity of 4.9 m/s. (e) Analysis of CMRI reveals a normal RV end-diastolic volume (76 ml), reduced RV ejection fraction of 28%, and preserved left ventricular ejection fraction of 60%. (f) CMRI scan shows subvalvular pulmonary stenosis (yellow arrow).

**Figure 3:**
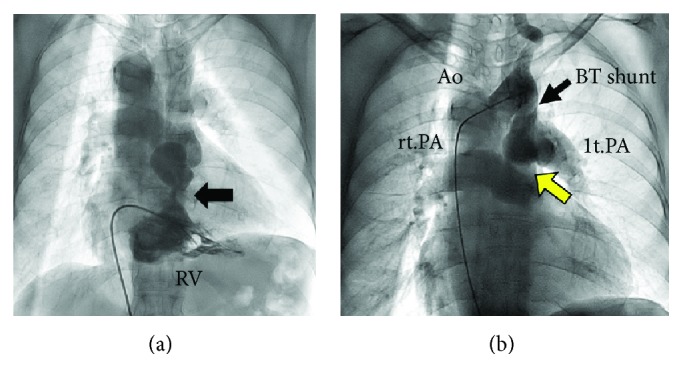
Catheter angiography. (a) Right ventriculogram shows subvalvular pulmonary stenosis (black arrow). (b) Aortogram shows a right-sided aortic arch and patent Blalock-Taussig shunt. There is stenosis at the ostium of the left pulmonary artery (yellow arrow) and both pulmonary artery aneurysms. Ao: Aorta; BT shunt; Blalock-Taussig shunt; lt.PA: left pulmonary artery; rt.PA: right pulmonary artery; RV: right ventricle.

At 70 years of age, she was found to have a low oxygen saturation during a health examination program, but she did not seek hospital care. She developed dyspnea and leg edema when she was 72 years old, and then diuretic was started for a diagnosis of heart failure at a local clinic. When she was referred to our hospital for further evaluation, her symptoms of heart failure were comparable with the New York Heart Association (NYHA) functional class III. Her oxygen saturation was 88% on room air at rest. Her heart examination revealed a continuous murmur (Levine III/VI) and systolic ejection murmur (IV/VI) in the second right sternal border. Laboratory data showed the following values (reference ranges): hemoglobin level 15.7 g/dl, hematocrit 44.5%, platelet count 19.7 × 10^4^/*μ*l, prothrombin time 11.4 seconds, 98.5%, D-dimer level 1.2 *μ*g/dl (<1.0 *μ*g/dl), creatinine level 1.35 mg/dl (0.4–0.8 mg/dl), and serum brain-type natriuretic peptide level 210 pg/ml (<18.4 pg/ml). A chest radiograph revealed a cardiothoracic ratio of 66%, right-sided aortic arch, and prominently dilated PA without pulmonary congestion (Figure 1(a)). The electrocardiogram showed sinus rhythm with a prolonged PR interval and ST-T segment abnormalities (Figure 1(b)). The echocardiogram showed a large ventricular septal defect with a bidirectional, mainly left-to-right shunt, overriding the aorta, right ventricular (RV) hypertrophy, and a stenotic RV outflow tract with a peak velocity of 4.9 m/s (Figure 2). Left ventricular (LV) diastolic indices of echocardiogram were as follows: peak E velocity 80 cm/s, E/A 0.72, septal E′ velocity 4.1 cm/s, lateral E′ velocity 3.5 cm/s, and average E/E′ 21. Abnormal relaxation pattern of transmitral flow, low E′ velocity, and high E/E′ suggested the existence of LV diastolic dysfunction. The cardiac magnetic resonance imaging scan showed reduced RV ejection fraction (EF) of 28% and preserved LVEF of 60% with a LV end-diastolic volume index of 103 ml/m^2^ (Figures 2(e) and 2(f)). There was no abnormality in the pulmonary valve (PV), and the diameter of the PV annulus was 24 mm. The catheter angiogram revealed a patent BTS and severe stenosis at the ostium of the left PA (Figure 3). Right heart catheterization showed a normal PA wedge pressure (systolic/diastolic/mean pressure: 15/9/8 mmHg), mildly elevated PA pressure (systolic/diastolic/mean pressure: 35/20/30 mmHg), elevated RV systolic and end-diastolic pressure (135/20 mmHg), mean right atrial pressure (11 mmHg), and systolic and end-diastolic LV pressure (149/23 mmHg). The pressure gradient between the PA and right ventricle was 117 mmHg. Cardiac output calculated by the Fick principle was 2.9 l/min, and pulmonary vascular resistance index was 5.95 Wood units·m^2^. The brain's magnetic resonance imaging scan showed no significant abnormalities. She started to take diuretics and home oxygen inspiration therapies; her heart failure symptom improved to NYHA II. Based on the clinical and hemodynamic assessments and her age, we decided not to perform radical operation.

## 3. Discussion

The patient was fortunate to have a patent right ventricular outflow tract (RVOT) that allowed her to live for ten years without surgery. The combination of the classic BTS at age 10 and the patent RVOT provided enough pulmonary blood flow to allow her to survive into her seventies. The use of the left classic BTS in the setting of a right aortic arch created a source of pulmonary blood flow that could grow along with her. Also, the RVOT must have grown along with her somatic growth [4].

Previous reports in PubMed of unoperated TOF surviving over 70 years old are shown in Table 1 [5–15]. There are rarely reported studies on long-term outcome about patients with TOF treated by only palliative operation.

Prolonged survival of patients with uncorrected TOF is often associated with a well-developed left ventricle, initially mild pulmonary stenosis, or well-maintained pulmonary blood flow by such as systemic to pulmonary collaterals or persistent patent ductus arteriosus. Furthermore, in our case, there was no history of significant organ dysfunction due to hypoxia, and no major complication of adult patients with uncorrected TOF such as brain abscess or infective endocarditis. Compared to previous reports (Table 1), it is an important thing that she gave birth to two children without significant symptoms of heart failure. In the case reported by Sousa et al. [15], the patient gave birth to one child without complications; however, her subpulmonary stenosis was very mild (maximum gradient of 31 mmHg) and her pulmonary blood flow was well maintained (Qp/Qs = 0.9). In our case, appropriately adjusted pulmonary blood flow by BTS might enable her to give birth to two children safely.

This patient had favorable anatomy that allowed her to live for a long time, but there was evidence of biventricular dysfunction accompanied with elevated right and left ventricular end-diastolic pressure. It might be caused by long-term pressure overload and cyanotic state. Although the diameter of the PV annulus was measured in her seventies, the size of 24 mm must have been acceptable for valve-sparing corrective surgery [16–18]. If she had undergone a valve-sparing corrective surgery in adolescence, much better quality of life and outcome could have been expected.

## 4. Conclusion

This case shows that classical BTS has a potential of long-term patency with good functional capacity. Appropriately adjusted pulmonary blood flow by BTS may cause long-term survival and a productive life in patients with TOF.

## Figures and Tables

**Table 1:** Previous reports in PubMed of unoperated TOF surviving over 70 years old.

Number	Author (year)	Age	Gender	Symptom or reason of admission	Other cardiac complications
1	Thomas et al. (1987) [5]	77	Male	Dyspnea	Ductus arteriosus, mitral regurgitation
2	Fernicola et al. (1993) [6]	74	Male	Dyspnea	Mitral prolapse
3	Ishida et al. (2001) [7]	71	Male	Dyspnea	None
4	Bielik et al. (2005) [8]	74	Male	Syncope	None
5	Tanaka et al. (2005) [9]	72	Female	Dyspnea	None
6	Yang et al. (2005) [10]	73	Male	Dyspnea	None
7	Alonso et al. (2007) [11]	86	Male	Chest pain	Coronary artery disease
8	Nieves et al. (2007) [12]	70	Female	Dyspnea	None
9	Stanescu et al. (2008) [13]	75	Male	Dyspnea	Quadricuspid aortic valve
10	Subhawoang et al. (2009) [14]	87	Female	Cerebral infraction	None
11	Sousa et al. (2013) [15]	72	Female	Dyspnea	Endocarditis
